# Supplementary material for: Structural basis of GTPase-mediated mitochondrial ribosome biogenesis and recycling
Source: Nat Commun. 2021 Jun 16;12:3672. doi: 10.1038/s41467-021-23702-y (PMC8209004; doi:10.1038/s41467-021-23702-y)
Supplement: Supplementary file 1 — Supplementary Information [file 41467_2021_23702_MOESM1_ESM.pdf]

Structural basis of GTPase-mediated mitochondrial ribosome biogenesis and recycling

Hauke S. Hillen[1,2,3,*], Elena Lavdovskaia[1,2], Franziska Nadler[1], Elisa Hanitsch[1], Andreas Linden[4,5], Katherine E. Bohnsack[6], Henning Urlaub[4,5] and Ricarda Richter-Dennerlein[1,2,*]

SUPPLEMENTARY DATA

Supplementary Figures

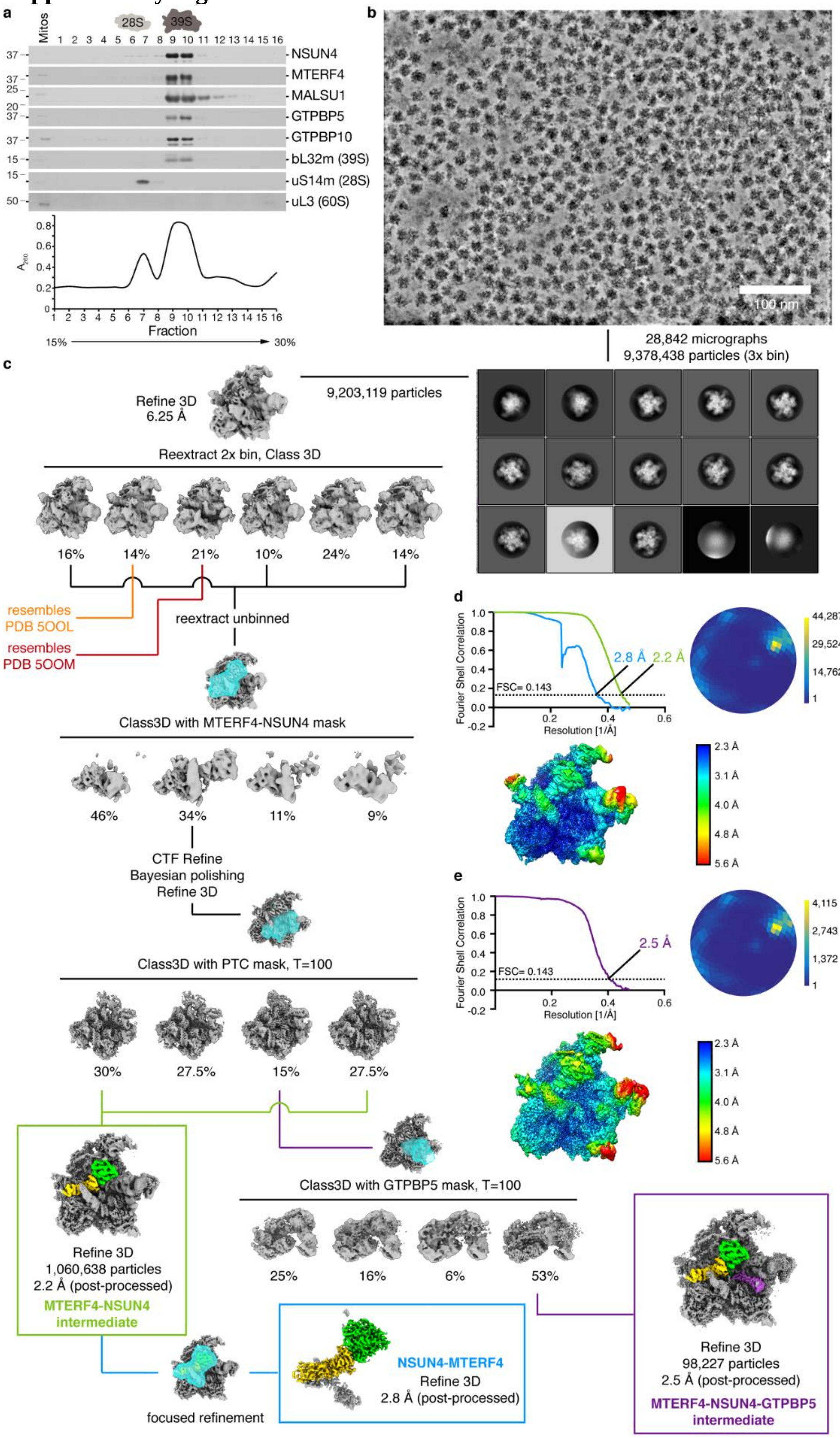

Supplementary Fig. 1 | Purification and Cryo EM data processing first dataset.
(a) Gradient fractions (1-16) upon ribosome isolation were analyzed by western blotting using indicated antibodies. uL3 (component of the cytosolic 60S large ribosomal subunit (LSU)) was used as a control to assess the level of contamination with cytosolic ribosomes. Absorbance was measured for each fraction at 260 nm. Fractions 8 and 9 were used for further analyses. The experiment was performed twice with reproducible outcome. Source data are provided as a Source Data file. **(b)** Example denoised micrograph calculated from two independently measured half sets of 40 frames each. Scale bar, 100 nm. **(c)** 2D class averages and Cryo-EM processing tree. **(d)** Fourier shell correlation (FSC) plot, Angular distribution plot and local resolution distribution for the MTERF4-NSUN4 large mitoribosomal subunit (mtLSU) intermediate. Scale for the angular distribution plot shows the number of particles assigned to a particular angular bin. Blue, a low number of particles; yellow, a high number of particles. **(e)** FSC plot, Angular distribution plot and local resolution distribution for the MTERF4-NSUN4-GTPBP5 mtLSU intermediate. Scale for the angular distribution plot shows the number of particles assigned to a particular angular bin. Blue, a low number of particles; yellow, a high number of particles.

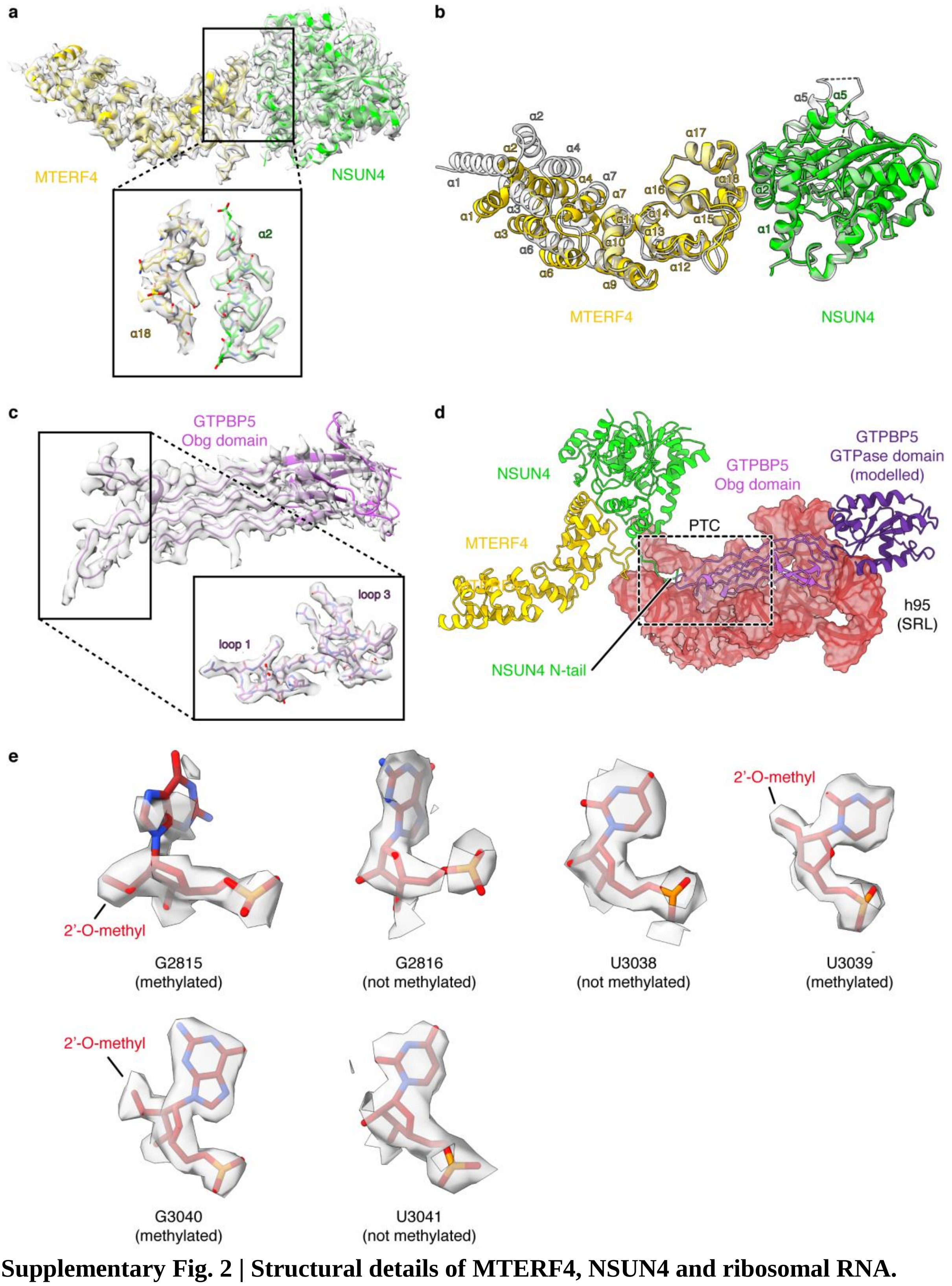

Supplementary Fig. 2 | Structural details of MTERF4, NSUN4 and ribosomal RNA.
(a) Density fit of MTERF4-NSUN4. The model of MTERF4-NSUN4 is shown as cartoon with coloring as in Fig. 1. The post-processed cryo-EM density from focused refinement is shown as transparent grey surface. The region at the interface between MTERF4 and NSUN4 is enlarged and shown as sticks. **(b)** Comparison between the MTERF4-NSUN4 complex bound to the large mitoribosomal subunit (mtLSU) and the previous crystal structure (PDB 4FP9) [15]. The two structures are shown superimposed as cartoon. The mtLSU-bound structure is colored

as in Fig. 1 and the free crystal structure is colored in grey and shown transparently. Secondary structure elements in MTERF4 and that adopt different conformations in NSUN4 are indicated. The helical repeats of MTERF4 form a widened curve when bound to the ribosome. **(c)** Density fit of GTPBP5. The model of GTPBP5 is shown as cartoon with coloring as in Fig. 2. The post-processed cryo-EM density is shown as transparent grey surface. The PTC-interacting loops described in Fig. 2 are shown enlarged as sticks. **(d)** Interaction of GTPBP5 with the mtLSU. Regions of the 16S rRNA interacting with MTERF4-NSUN4 are shown as cartoon and as transparent surface. MTERF4, NSUN4 and GTPBP5 are shown as cartoons. SRL: sarcin-ricin loop. **(e)** G2815, U3039 and G3040 are methylated. G2815, U3039 and G3040 are shown as sticks with the post-processed cryo-EM density of the MTERF4-NSUN4-GTPBP5 mtLSU intermediate (dataset 1) shown as transparent surface. Neighboring non-methylated bases are shown for comparison.

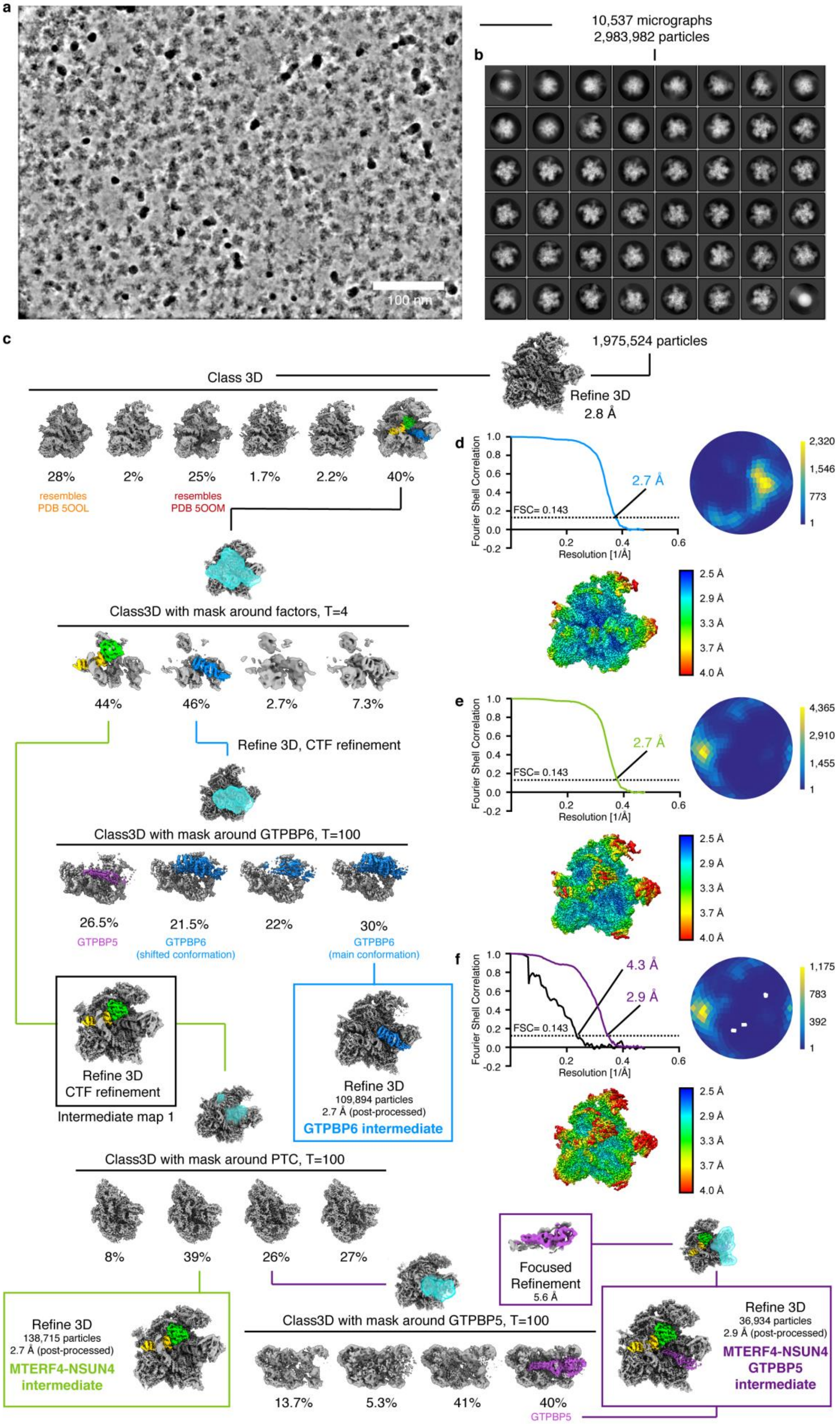
a
100 nm
10,537 micrographs
2,983,982 particles
b
c
1,975,524 particles
Refine 3D
2.8 Å
Class 3D
28%
2%
25%
1.7%
2.2%
40%
resembles PDB 5OOL
resembles PDB 5OOM
Class3D with mask around factors, T=4
44%
46%
2.7%
7.3%
Refine 3D, CTF refinement
Class3D with mask around GTPBP6, T=100
26.5%
21.5%
22%
30%
GTPBP5
GTPBP6 (shifted conformation)
GTPBP6 (main conformation)
Refine 3D
CTF refinement
Intermediate map 1
Refine 3D
109,894 particles
2.7 Å (post-processed)
GTPBP6 intermediate
Class3D with mask around PTC, T=100
8%
39%
26%
27%
Refine 3D
138,715 particles
2.7 Å (post-processed)
MTERF4-NSUN4 intermediate
Class3D with mask around GTPBP5, T=100
13.7%
5.3%
41%
40%
GTPBP5
Focused Refinement
5.6 Å
Refine 3D
36,934 particles
2.9 Å (post-processed)
MTERF4-NSUN4 GTPBP5 intermediate
d
e
f
Fourier Shell Correlation
Resolution [1/Å]
FSC= 0.143
2.7 Å
4.3 Å
2.9 Å
2,320
1,546
773
4,365
2,910
1,455
1,175
783
392
2.5 Å
2.9 Å
3.3 Å
3.7 Å
4.0 Å

Supplementary Fig. 3 | Cryo-EM data processing second dataset.
(a) Example denoised micrograph calculated from two independently measured half sets of 40 frames each. Scale bar, 100 nm. **(b)** 2D class averages. **(c)** Cryo-EM processing tree. **(d)** Fourier shell correlation (FSC) plot, Angular distribution plot and local resolution distribution for the GTPBP6-bound large mitoribosomal subunit (mtLSU) intermediate. Scale for the angular distribution plot shows the number of particles assigned to a particular angular bin. Blue, a low number of particles; yellow, a high number of particles. **(e)** Fourier shell correlation (FSC) plot, Angular distribution plot and local resolution distribution for the MTERF4-NSUN4 mtLSU intermediate. Scale for the angular distribution plot shows the number of particles assigned to a particular angular bin. Blue, a low number of particles; yellow, a high number of particles. **(f)** FSC plot, Angular distribution plot and local resolution distribution for the MTERF4-NSUN4-GTPBP5 mtLSU intermediate. Scale for the angular distribution plot shows the number of particles assigned to a particular angular bin. Blue, a low number of particles; yellow, a high number of particles.

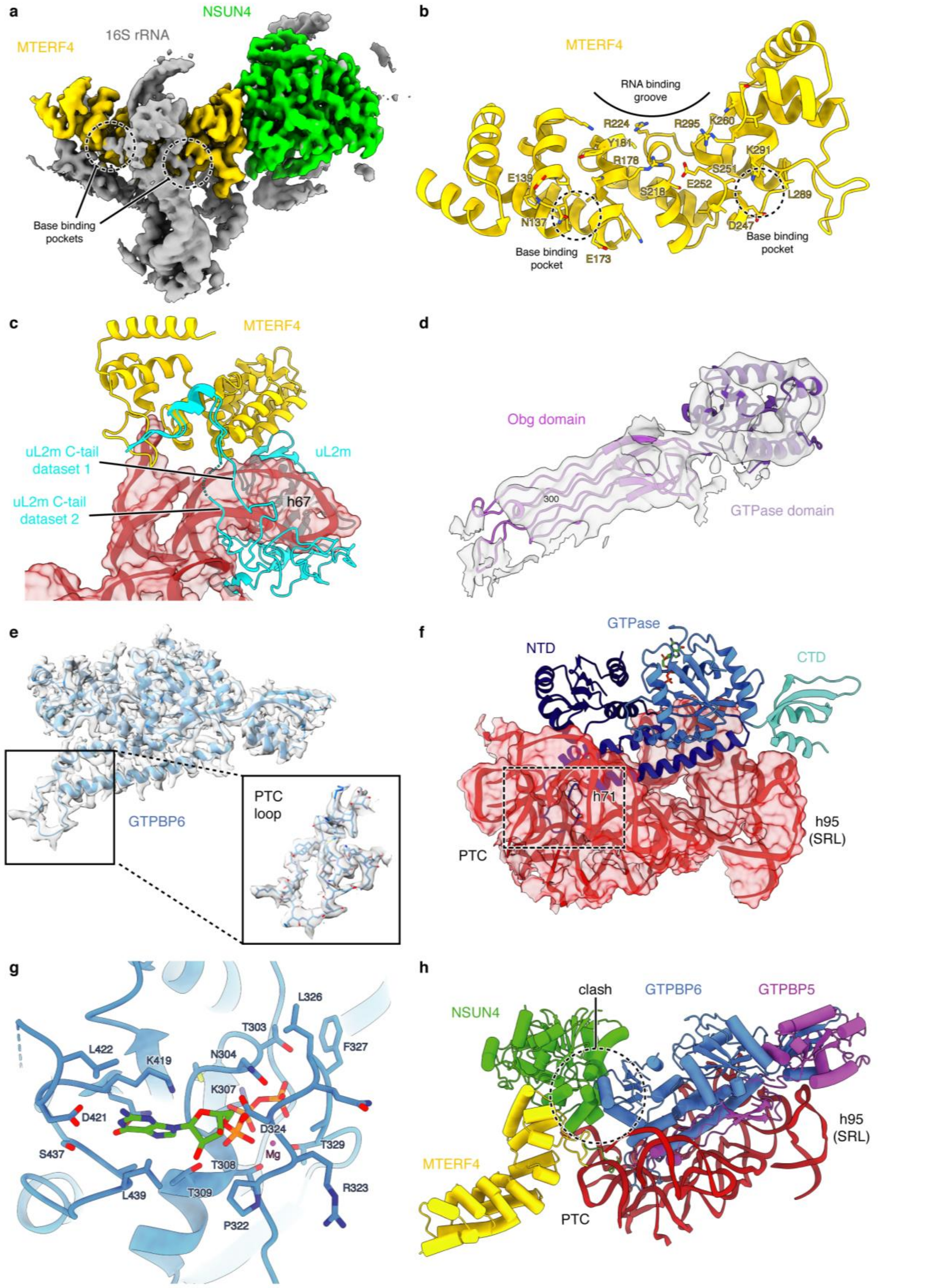

Supplementary Fig. 4 | Structural details of MTERF4, NSUN4, GTPBP5 and GTPBP6 in the second dataset.

(a) Improved density for the 16 rRNA region wrapping over MTERF4. A region of the cryo-EM reconstruction (Intermediate map 1, Supplementary Fig. 3) of MTERF4-NSUN4

containing particles from dataset 2 is shown as surface and colored as follows: NSUN4: lime green, MTERF4: yellow, 16S rRNA: grey. The region of the rRNA wrapping above MTERF4 adopts a helical fold and may form base-mediated interactions with MTERF4 (indicated). **(b)** Close-up view of the RNA-binding groove of MTERF4. MTERF4 is shown as cartoon in yellow and residues close to the RNA density observed in (a) are shown as sticks. The potential base-binding pockets are indicated. **(c)** The C-tail of uL2m occupies different paths in the MTERF4-NSUN4 mtLSU intermediate from dataset 1 and dataset 2. MTERF4 and uL2m are shown as cartoons and the 16S rRNA elements interacting with them are shown as cartoon and transparent surface. Coloring as in Fig. 1. **(d)** Improved density for the GTPBP5 GTPase domain in dataset 2. GTPBP5 is shown as cartoon and colored as in Fig. 2. The cryo-EM density obtained from focused refinement of the GTPBP5-containing particle set in dataset 2 is shown as transparent grey surface. **(e)** Density fit of GTPBP6. GTPBP6 is shown as cartoon and colored in blue. The post-processed cryo-EM density of the GTPBP6-bound mtLSU intermediate structure is shown as transparent grey surface. The PTC loop described in Fig. 3 is shown enlarged as sticks. **(f)** Interaction of GTPBP6 with the mtLSU. Regions of the 16S rRNA interacting with GTPBP6 are shown as cartoon and as transparent surface. GTPBP6 is shown as cartoon. **(g)** GTP binding pocket of GTPBP6. The GTP binding site of GTPBP6 is shown as cartoon and colored as in Fig. 3. Residues within 4 Å of GTP are shown as sticks. GTP is shown in green as sticks. **(h)** GTPBP6 occupies the same binding site as GTPBP5 on the mtLSU and would clash with NSUN4. The structure of the MTERF4-NSUN4-GTPBP5 intermediate and the GTPBP6 intermediate were superimposed with their 16S rRNA. The peptidyl transferase center (PTC) and factors are shown as cartoons and colored as in Fig. 1-3. SRL: sarcin-ricin loop.

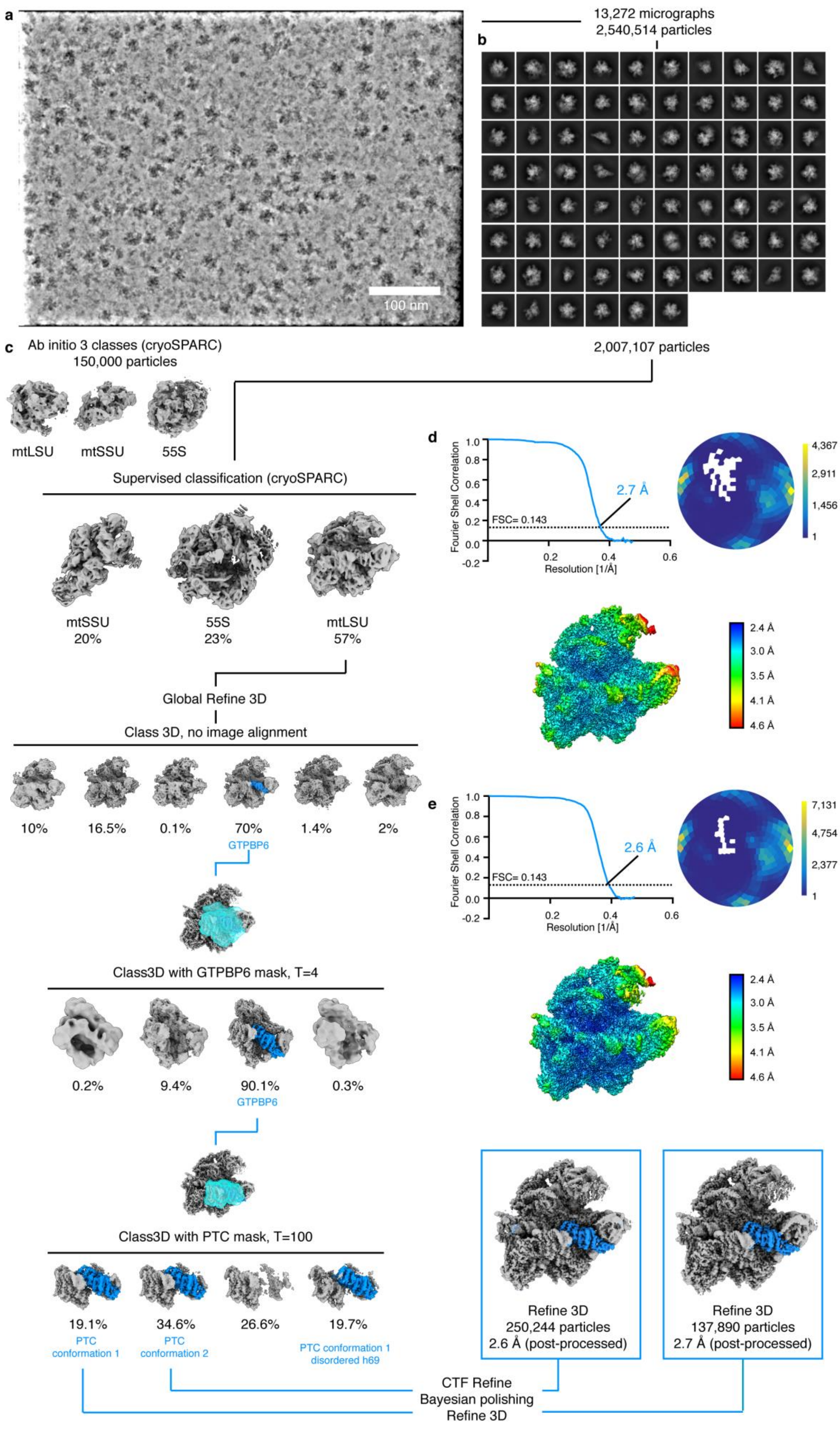
a
100 nm
13,272 micrographs
2,540,514 particles
b
c
Ab initio 3 classes (cryoSPARC)
150,000 particles
2,007,107 particles
mtLSU
mtSSU
55S
Supervised classification (cryoSPARC)
mtSSU
20%
55S
23%
mtLSU
57%
Global Refine 3D
Class 3D, no image alignment
10%
16.5%
0.1%
70%
GTPBP6
1.4%
2%
Class3D with GTPBP6 mask, T=4
0.2%
9.4%
90.1%
GTPBP6
0.3%
Class3D with PTC mask, T=100
19.1%
PTC conformation 1
34.6%
PTC conformation 2
26.6%
19.7%
PTC conformation 1 disordered h69
d
Fourier Shell Correlation
Resolution [1/Å]
FSC= 0.143
2.7 Å
4,367
2,911
1,456
1
2.4 Å
3.0 Å
3.5 Å
4.1 Å
4.6 Å
e
2.6 Å
7,131
4,754
2,377
Refine 3D
250,244 particles
2.6 Å (post-processed)
Refine 3D
137,890 particles
2.7 Å (post-processed)
CTF Refine
Bayesian polishing
Refine 3D

Supplementary Fig. 5 | Cryo-EM data processing third dataset.
(a) Example denoised micrograph calculated from two independently measured half sets of 40 frames each. Scale bar, 100 nm. **(b)** 2D class averages. **(c)** Cryo-EM processing tree. **(d)** Fourier shell correlation (FSC) plot, Angular distribution plot and local resolution distribution for the GTPBP6-bound split large mitoribosomal subunit (mtLSU) with peptidyl transferase center (PTC) conformation 1. Scale for the angular distribution plot shows the number of particles assigned to a particular angular bin. Blue, a low number of particles; yellow, a high number of particles. **(e)** FSC plot, Angular distribution plot and local resolution distribution for the GTPBP6-bound split mtLSU with PTC conformation 2. Scale for the angular distribution plot shows the number of particles assigned to a particular angular bin. Blue, a low number of particles; yellow, a high number of particles.

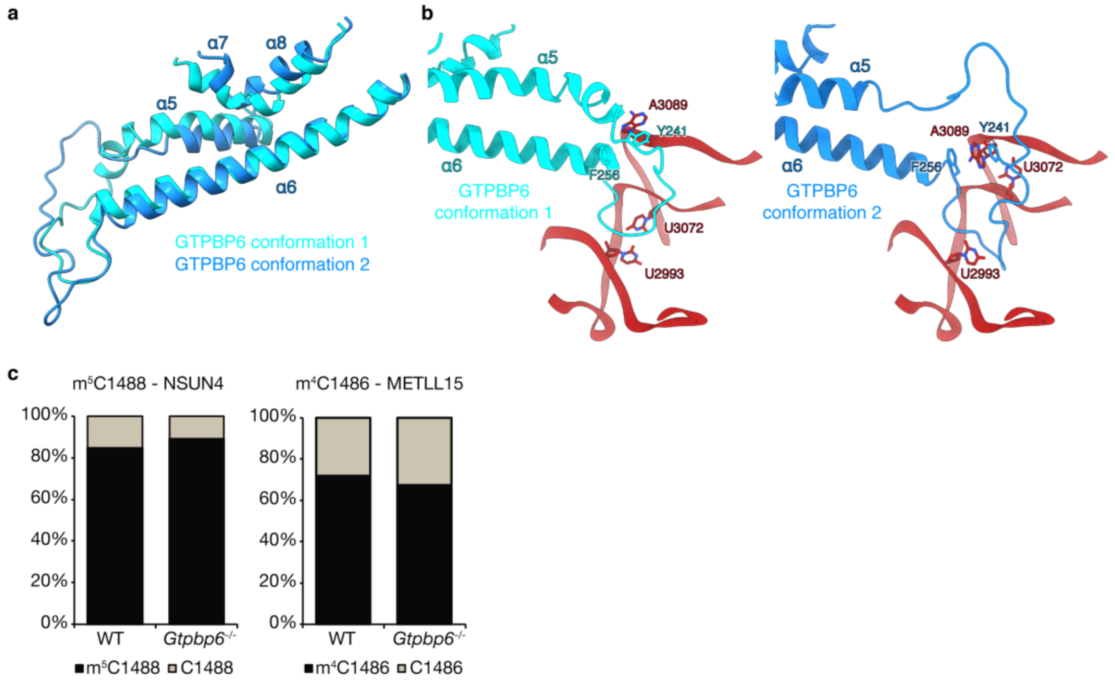

Supplementary Fig. 6 | GTPBP6 conformations.

(a) The PTC-binding loop of GTPBP6 can adopt two conformations. Superimposition of GTPBP6 in the PTC conformation 1 and PTC conformation 2 structures observed after ribosome splitting (dataset 3). GTPBP6 is shown as cartoon. GTPBP6 conformation 1, which is also observed during mtLSU biogenesis, is shown in cyan and GTPBP6 conformation 2 in marine. **(b)** Close-up of PTC interactions in GTPBP6 PTC conformation 1 and 2. Depiction as in a, with the region of the 16S rRNA differing between the two states shown as cartoon. Bases that adopt different conformations as well as interacting GTPBP6 residues are shown as sticks. **(c)** Methylation of 12S rRNA is not affected in GTPBP6-deficient cells. Total RNA from wild type (WT) or *Gtpbp6*$^{-/-}$ was treated with bisulfite, reverse transcribed and a region of the 16S rRNA containing m^{5}C1488 and m^{4}C1486 was amplified, cloned and sequenced. The relative proportions of unconverted cytosine reflecting m^{5}C/m^{4}C (black) and thymine reflecting converted, unmodified cytosine (grey) are shown. Data derived from sequencing of 46 individual clones per sample are presented. Source data are provided as a Source Data file.

Supplementary Tables

Supplementary Table 1 | Cryo-EM data collection and refinement for dataset 1.

	**MTERF4-NSUN4 intermediate (PDB: 7OF0)**	**MTERF4-NSUN4-GTPBP5-intermediate (PDB: 7OF7)**
Data collection and processing		
Magnification	81,000	
Voltage (kV)	300	
Electron exposure (e–/Å^2)	36	
Defocus range (μm)	0.3 – 2.8	
Pixel size (Å)	1.05	
Symmetry imposed	C1	
Initial particle images (no.)	9,378,438	
Final particle images (no.)	1,060,638	98,227
Map resolution (Å)	2.2	2.5
FSC threshold	0.143	0.143
Map sharpening *B* factor (Å^2)	-50	-39.5
Map resolution range (Å)	5.6 – 2.2	5.6 – 2.2
Refinement		
Model resolution (Å)	2.3	2.7
FSC threshold	0.5	0.5
Model composition		
Non-hydrogen atoms	99,476	101,032
Protein/Nucleotide residues	8,446 / 1,432	8,655 / 1,432
Ligands	ZN: 3, MG: 61	ZN:3, MG:74
B factors (Å^2)		
Protein/Nucleotide	107.39 / 97.44	94.53 / 71.94
Ligand	62.66	46.80
R.m.s. deviations		
Bond lengths (Å)	0.006	0.006
Bond angles (°)	0.859	0.874
Validation		
MolProbity score	1.54	1.52
Clashscore	7.28	5.58
Poor rotamers (%)	0.00	0.00
Ramachandran plot		
Favored (%)	97.26	96.61
Allowed (%)	2.74	3.39
Disallowed (%)	0.00	0.00

Supplementary Table 2 | Cryo-EM data collection and refinement for dataset 2.

	GTPBP6 intermediate (PDB: 7OF2)	MTERF4-NSUN4 intermediate (PDB: 7OF3)	MTERF4-NSUN4-GTPBP5-intermediate (PDB: 7OF5)
Data collection and processing			
Magnification		81,000	
Voltage (kV)		300	
Electron exposure (e–/Å^2)		37	
Defocus range (μm)		0.3 – 2.1	
Pixel size (Å)		1.05	
Symmetry imposed		C1	
Initial particle images (no.)		2,983,982	
Final particle images (no.)	109,894	138,715	36,934
Map resolution (Å)	2.7	2.7	2.9
FSC threshold	0.143	0.143	0.143
Map sharpening *B* factor (Å^2)	-52	-58	-53
Map resolution range (Å)	4.0 – 2.5	4.0 – 2.5	4.0 – 2.5
Refinement			
Model resolution (Å)	2.8	2.9	3.0
FSC threshold	0.5	0.5	0.5
Model composition			
Non-hydrogen atoms	101,195	101,226	102,785
Protein/Nucleotide residues	8,560 / 1,466	8,627 / 1,442	8,829 / 1,445
Ligands	GTP: 3, ZN: 3, MG: 85	GTP: 2, ZN: 3, MG: 58	GTP: 2, ZN: 3, MG: 70
B factors (Å^2)			
Protein/Nucleotide	84.56 / 65.75	94.62 / 76.45	102.28 / 84.03
Ligand	59.47	69.93	72.24
R.m.s. deviations			
Bond lengths (Å)	0.004	0.04	0.004
Bond angles (°)	0.815	0.804	0.812
Validation			
MolProbity score	1.39	1.50	1.45
Clashscore	4.30	5.64	4.93
Poor rotamers (%)	0.01	0.00	0.00
Ramachandran plot			
Favored (%)	96.93	96.81	96.83
Allowed (%)	3.07	3.18	3.17
Disallowed (%)	0.00	0.01	0.00

Supplementary Table 3 | Cryo-EM data collection and refinement for dataset 3.

	**Mature mtLSU with GTPBP6 – PTC state 1 (PDB: 7OF4)**	**Mature mtLSU with GTPBP6 – PTC state 2 (PDB: 7OF6)**
Data collection and processing		
Magnification	81,000	
Voltage (kV)	30	
Electron exposure (e–/Å^2)	40	
Defocus range (μm)	0.2 – 2.7	
Pixel size (Å)	1.05	
Symmetry imposed	C1	
Initial particle images (no.)	2,540,514	
Final particle images (no.)	137,890	250,244
Map resolution (Å)	2.7	2.6
FSC threshold	0.143	0.143
Map sharpening *B* factor (Å^2)	-57	-66
Map resolution range (Å)	4.6 – 2.4	4.6 – 2.4
Refinement		
Model resolution (Å)	2.8	2.7
FSC threshold	0.5	0.5
Model composition		
Non-hydrogen atoms	100,322	100,321
Protein/Nucleotide residues	8,320 / 1,519	8,320 / 1,519
Ligands	GTP: 3, ZN: 3, MG: 89	GTP:3, ZN: 3, MG: 88
B factors (Å^2)		
Protein/Nucleotide	92.56 / 73.81	86.71 / 69.37
Ligand	70.84	65.35
R.m.s. deviations		
Bond lengths (Å)	0.005	0.003
Bond angles (°)	0.818	0.805
Validation		
MolProbity score	1.45	1.38
Clashscore	4.92	4.18
Poor rotamers (%)	0.00	0.01
Ramachandran plot		
Favored (%)	96.83	96.99
Allowed (%)	3.17	3.01
Disallowed (%)	0.00	0.00

Supplementary Table 4 | Primers

**Primer**	**Sequence**
12S-m^{5}C841_RT	5’-TTTAATTAAATATCCTTTAAAATATAC-3’
12S forward	5’-TTTAATTAAATATCCTTTAAAATATAC-3’
12S reverse	5’-AATAGGGTTTTGAAGTGTGTATATA-3’
GTPBP6 LIC for	5’-TACTTCCAATCCAATGCAcccgggaatctggaggggcc-3’
GTPBP6 LIC rev	5’-TTATCCACTTCCAATGTTATTAtcctggaaagagcttccggaatttgcc-3’
