# Supplementary material for: Structural basis of GTPase-mediated mitochondrial ribosome biogenesis and recycling
Source: Nat Commun. 2021 Jun 16;12:3672. doi: 10.1038/s41467-021-23702-y (PMC8209004; doi:10.1038/s41467-021-23702-y)
Supplement: Supplementary file 5 — Description of Additional Supplementary Files [file 41467_2021_23702_MOESM5_ESM.docx]

Description of additional supplementary information

Title: Supplementary Table 1

Description: LC-MS/MS analysis of mtLSU and mtSSU purified from GTPBP6-deficient cells. (Excel file)

Title: Supplementary Movie 1

Description: Model of peptidyl transferase center maturation during human mitochondrial ribosome biogenesis
