# Supplementary figures and images for: Structural basis of GTPase-mediated mitochondrial ribosome biogenesis and recycling
Source: Nat Commun. 2021 Jun 16;12:3672. doi: 10.1038/s41467-021-23702-y (PMC8209004; doi:10.1038/s41467-021-23702-y)

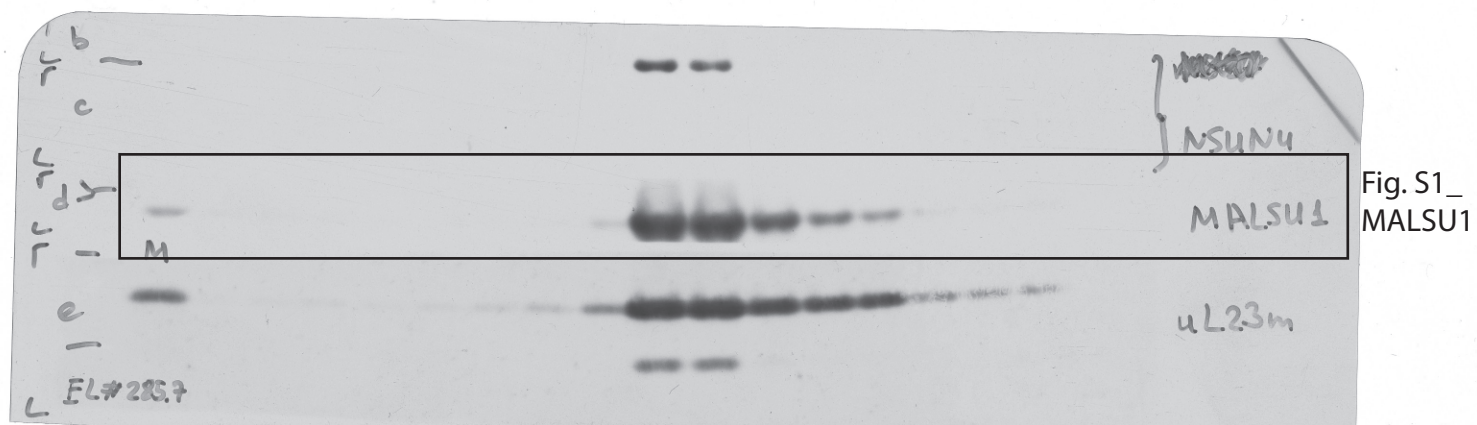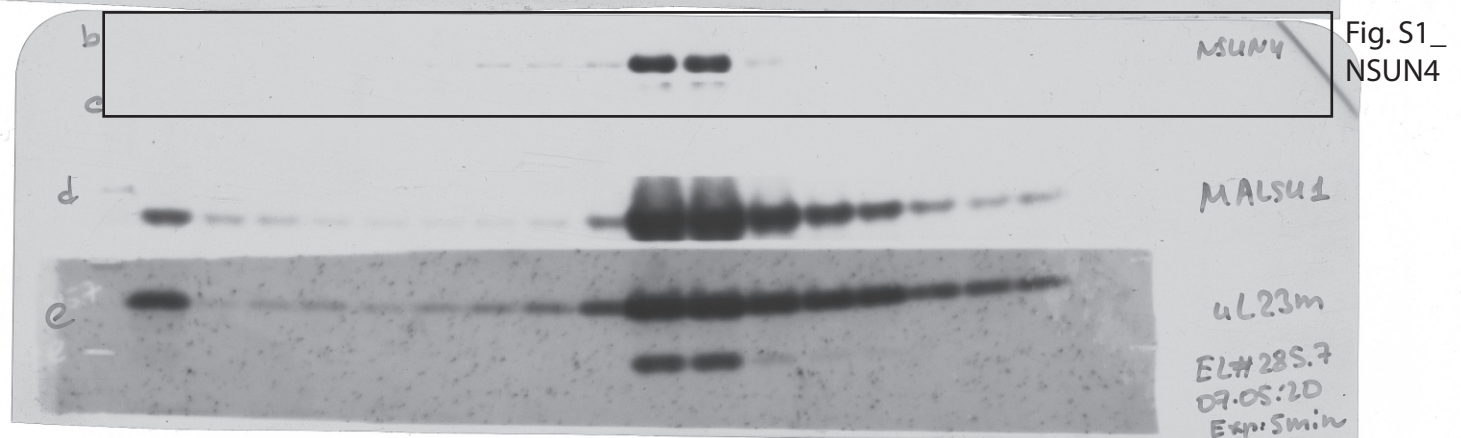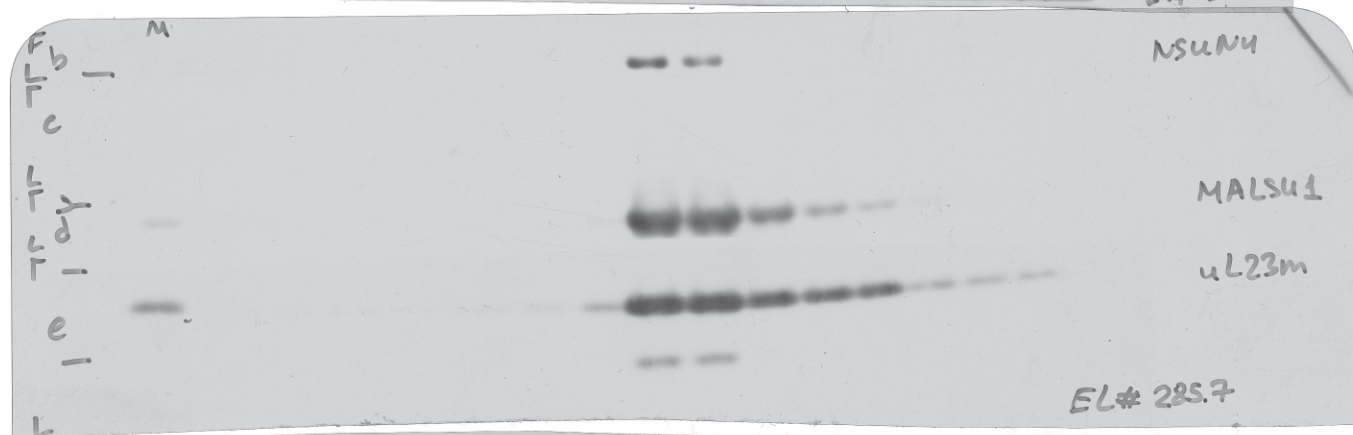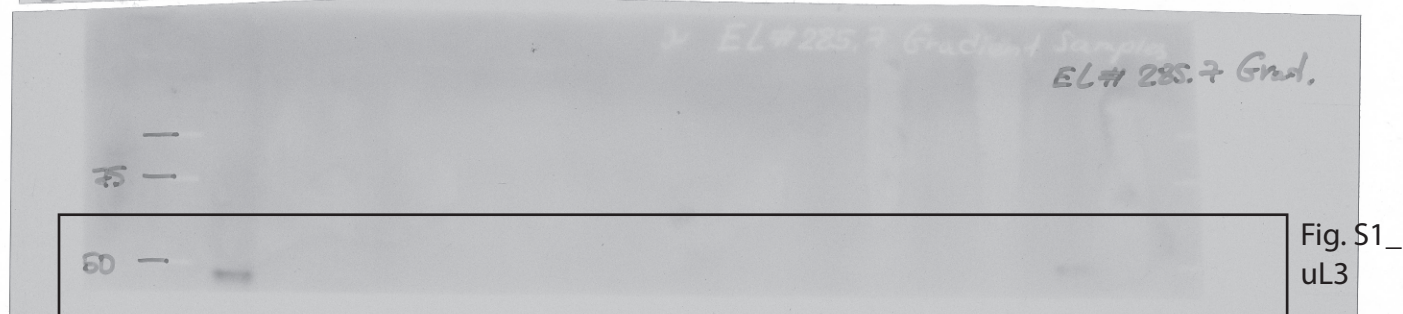

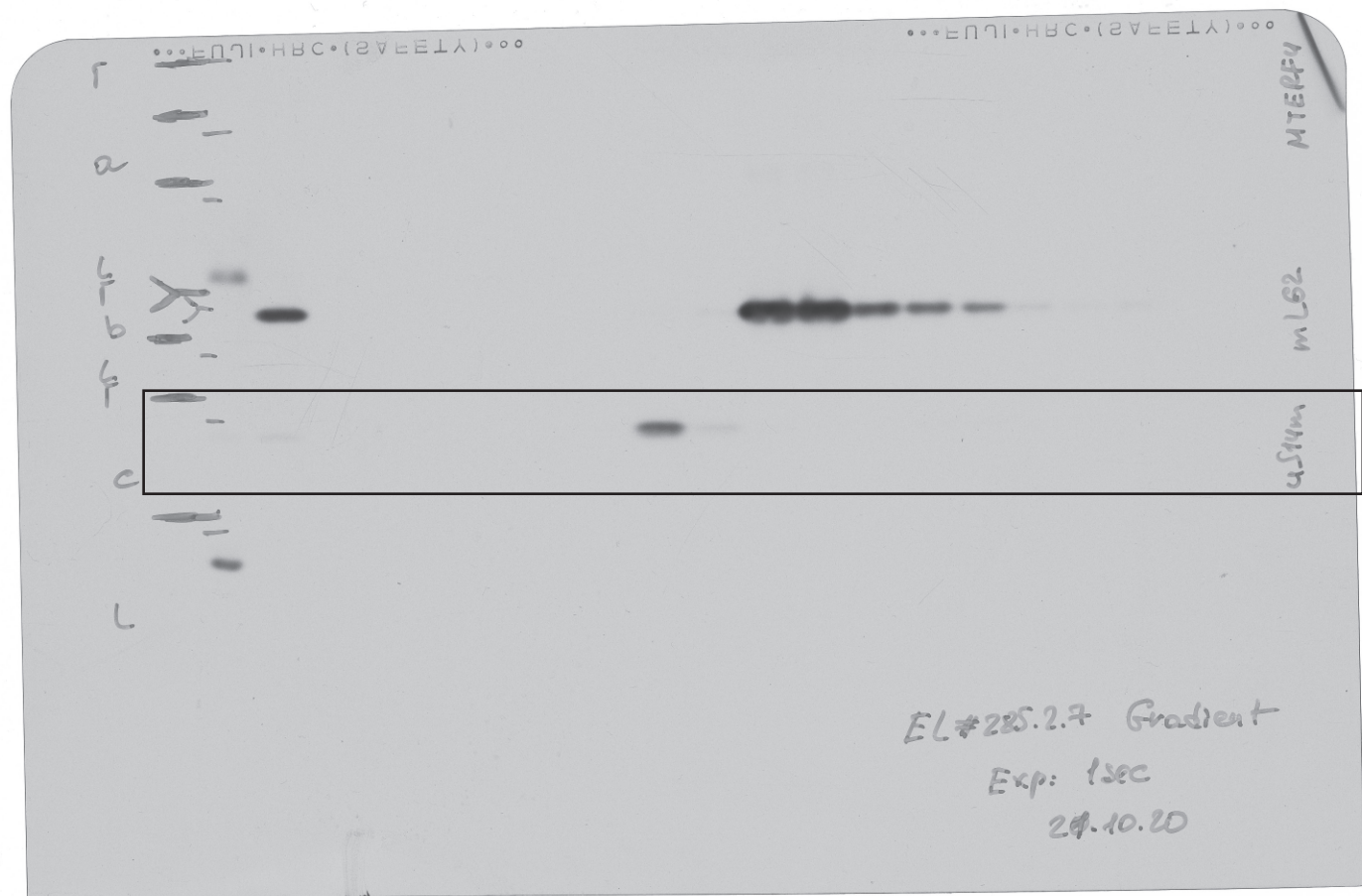

Fig. S1\_uS14m

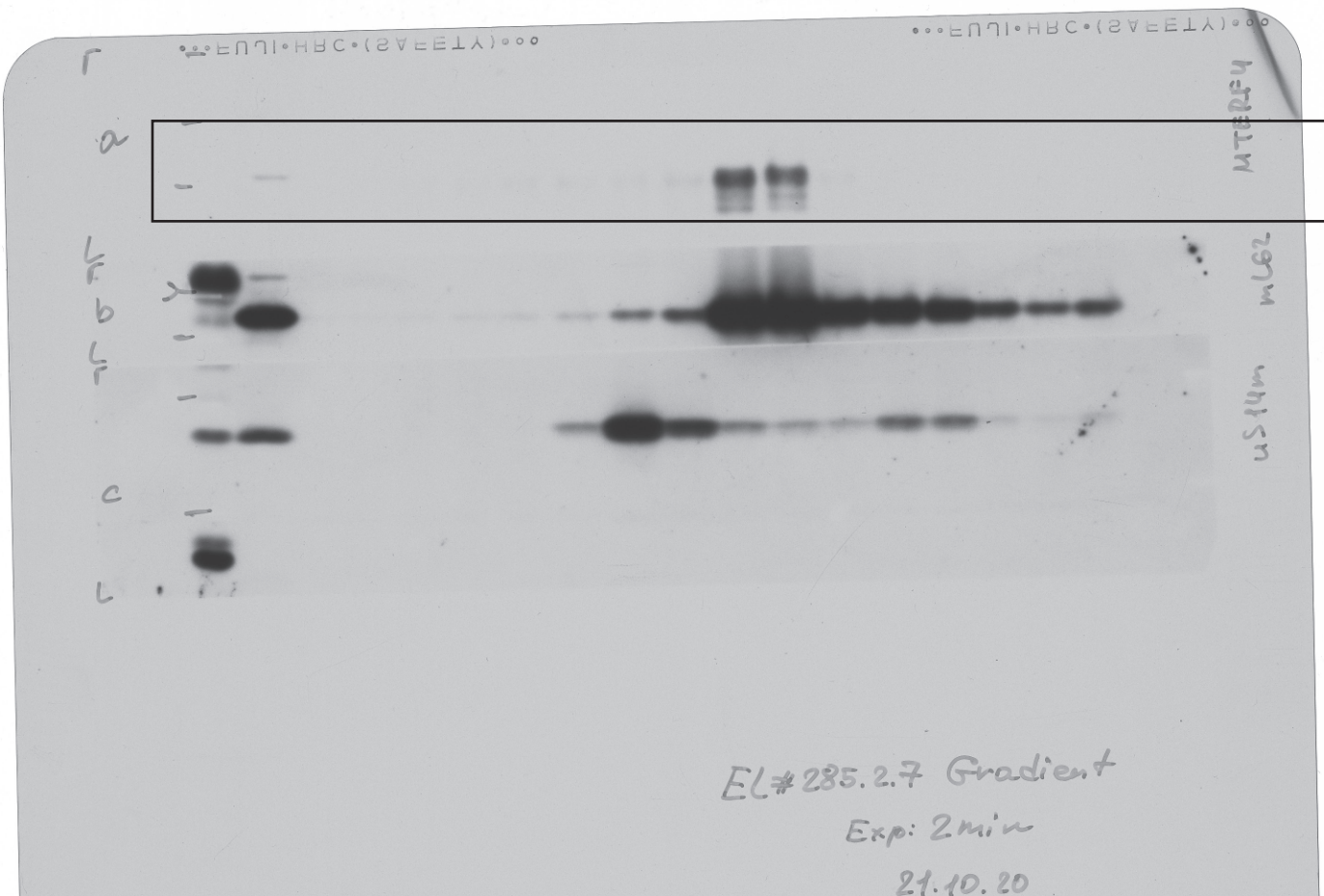

Fig. S1\_MTERF4

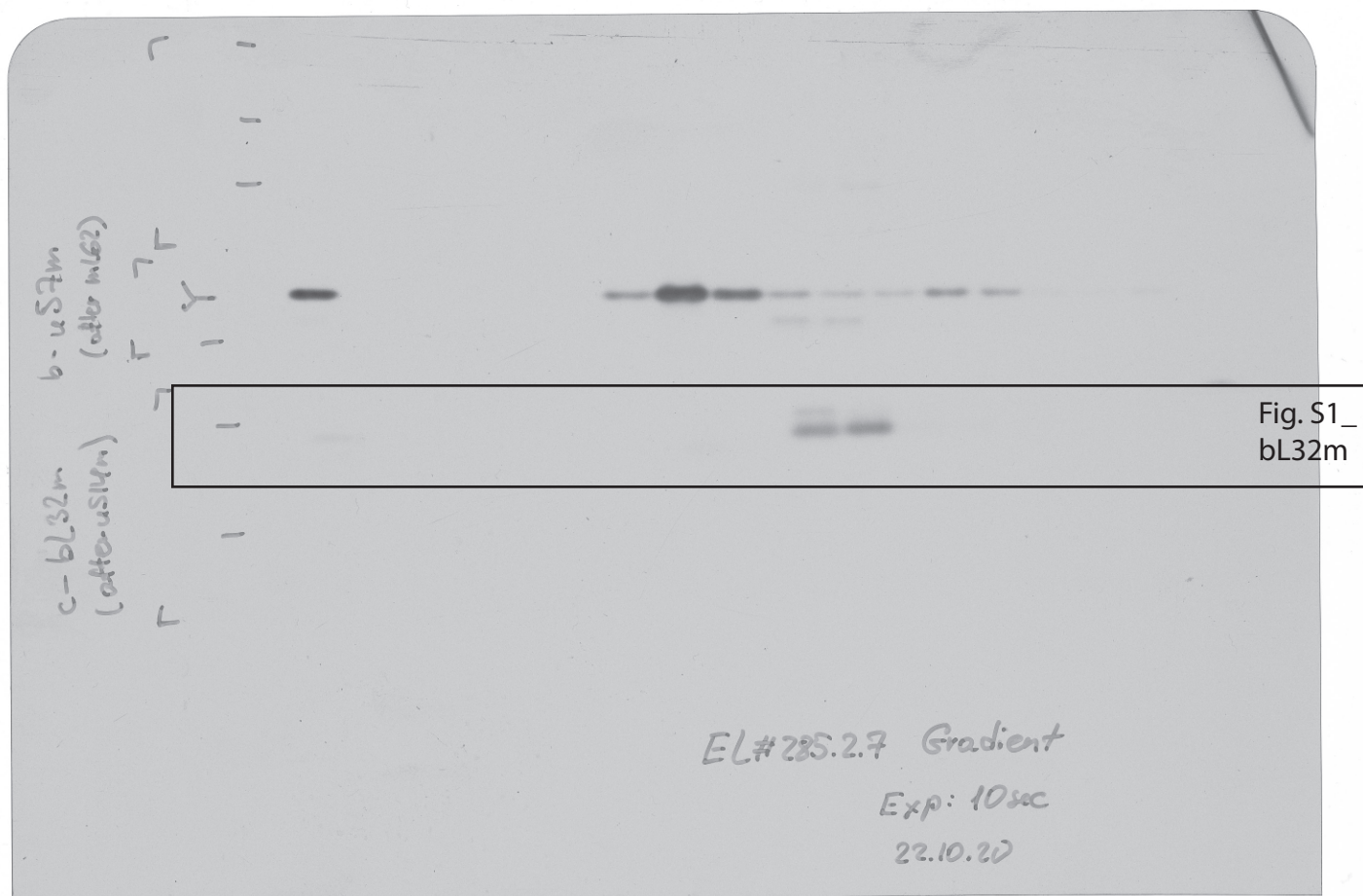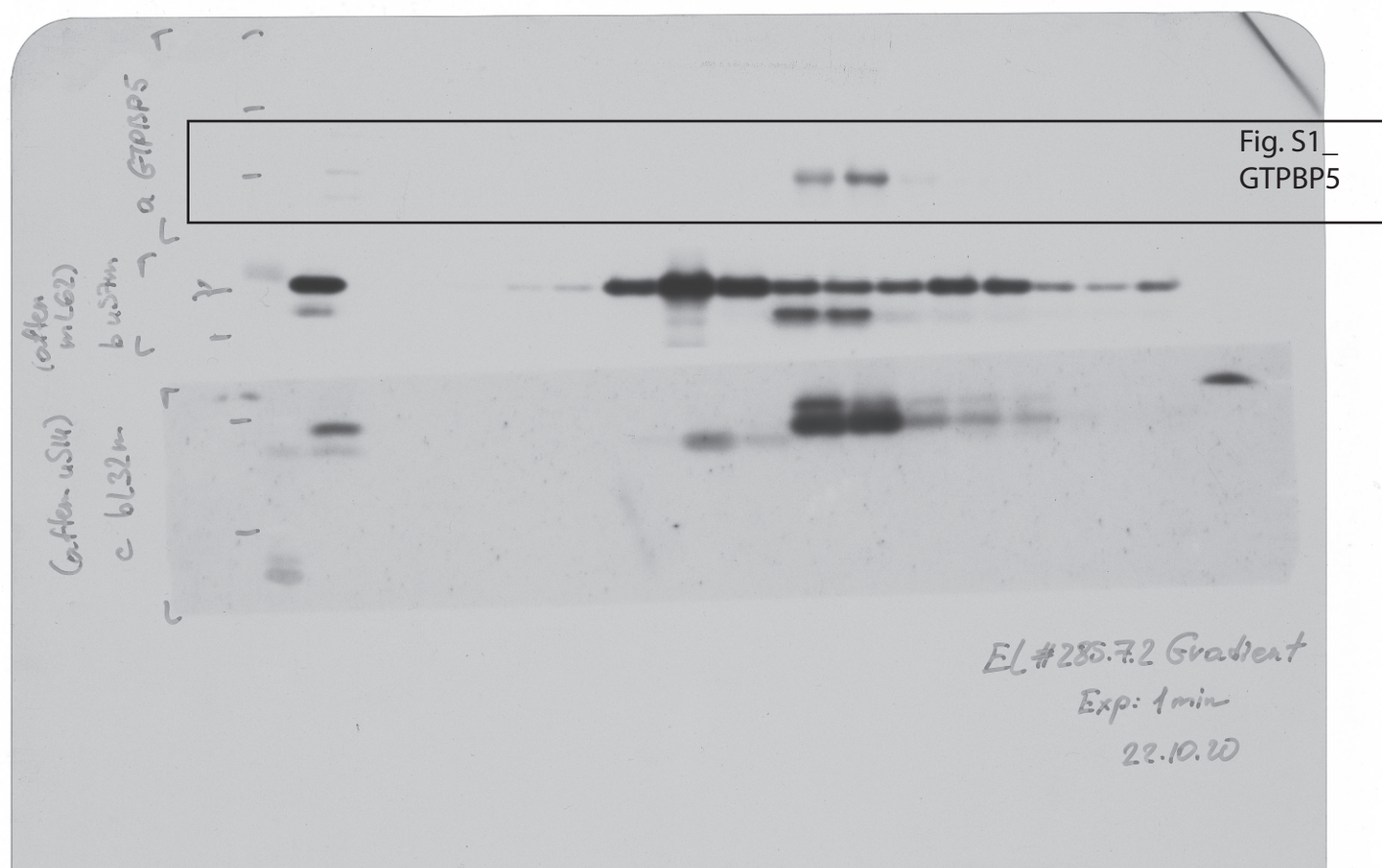

2

Gradient!

EL# 29512

AD: GTPBP10  
1:1000

Exp: 5min

29.07.20

Fig. S1\_  
GTPBP10

ETY)...

...FUJI-HRC-(SAFETY)...

...F

Supplement: Supplementary file 7 — Source Data [file 41467_2021_23702_MOESM7_ESM.zip › Source Data Supplementary Figure 1.pdf]
